# Edge-Up Oriented Colloidal CdSe Nanoplatelets Facilitate Faster Response in Vertical Photodetectors

**DOI:** 10.1021/acsami.6c04330

**Published:** 2026-06-29

**Authors:** Mohammed A. Ibrahem, Mohsin Waris, Farzan Shabani, Md Rumon Miah, Emre Unal, Betul Canimkurbey, Savas Delikanli, Hilmi Volkan Demir

**Affiliations:** † Department of Electrical and Electronics Engineering, Department of Physics, UNAM−Institute of Materials Science and Nanotechnology, and the National Nanotechnology Research Center, Bilkent University, Ankara 06800, Türkiye; ‡ Laser Science and Technology Department, College of Applied Sciences, 226156 University of Technology, Baghdad 10066, Iraq; § Applied Sciences Research Unit, College of Applied Sciences, University of Technology, Baghdad 10066, Iraq; ∥ Department of Physics, Faculty of Polatlı Science and Art, Ankara Hacı Bayram Veli University, Ankara 06900, Türkiye; ⊥ Luminous! Center of Excellence for Semiconductor Lighting and Displays, School of Electrical and Electronic Engineering, Division of Physics and Applied Physics, School of Physical and Mathematical Sciences, School of Materials Science and Engineering, 54761 Nanyang Technological University, Singapore 639798, Singapore; # Yildiz Technical University, Department of Metallurgical and Materials Engineering, Istanbul 34220, Türkiye

**Keywords:** colloidal nanocrystals, colloidal quantum wells, MSM photodetector, self-assembly orientation control, ligand exchange

## Abstract

Integrating self-assembled colloidal nanocrystals with uniform orientation into optoelectronic devices may allow for the significant improvement of charge transport processes. In this work, for this purpose, using the liquid–air interface self-assembly technique and selectively controlling the orientation of CdSe nanoplatelets (NPLs) with short 2-ethylhexane-1-thiol (EHT) ligands in a vertical-configuration photodetector device, we show that the photoconductivity response is substantially improved by the selective arrangement of CdSe NPLs into two distinct assemblies of edge-up (EO) and face-down (FO) orientations compared to the randomly oriented (RO) NPL film deposited by spin-coating. Our devices reveal that EO nanoplatelets significantly enhance response speed, while RO yields higher photocurrent, responsivity and detectivity. The assembled devices, consisting of one-monolayer EO and three-monolayer FO NPL films of comparable vertical film thickness, demonstrate superior photocurrent responses of 8 ms/11.3, 13 ms/4.5, and 15.2 ms for the rise/decay time constants, respectively, compared to 17 ms/18, and 80 ms for the RO for the rise/decay time constants. Despite using only a monolayer of EO NPLs, we achieved a responsivity of 21.04 mAW^–1^ and a detectivity of 5.77 × 10^10^ Jones, compared with the best results from CdSe-based photodetectors reported in the literature. This work provides critical insight for charge transportation management in solution-processed photodetection devices by adjusting the orientation of the two-dimensional quantum structures, paving the way toward fast and atomically thin functional optoelectronic devices. This also demonstrates that facet-specific metal–semiconductor interfaces are another critical factor, in addition to the charge transportation pathway, which can modulate the interfacial electronic structure and recombination dynamics in vertical configuration photodetectors.

## Graphical abstract

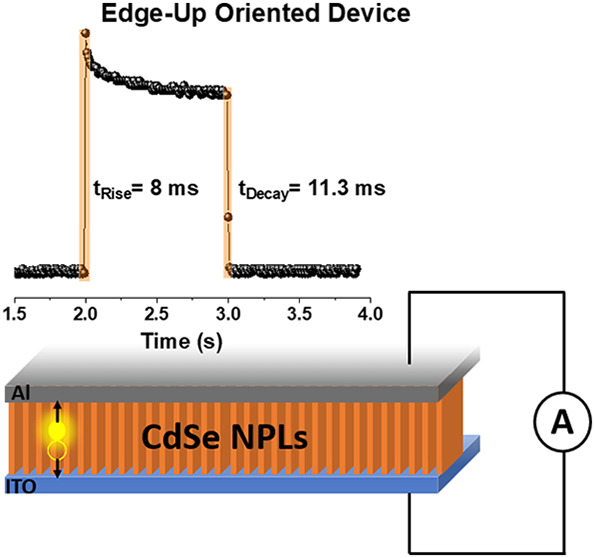

## 1 Introduction

Since the emergence of the two-dimensional colloidal quantum wells (2D-CQWs), also known as nanoplatelets (NPLs),^1,2^ their superior light emission properties, owing to their atomically uniform thickness and pronounced vertical quantum confinement, have been widely investigated.^3^ The advances in synthetic methods, which resulted in a vast array of NPLs with different properties, have paved the way for utilizing these quantum emitters in the new generation of optoelectronic devices with innovative designs and optimizations.^4–7^ Light-emitting diodes (LEDs) and lasers are the leading applications that have benefited the most from the semiconductor NPLs, thanks to their high photoluminescence quantum yield and high optical gain.^8^ Among the cadmium chalcogenide CQWs, CdSe-based heterostructures have also been most rigorously studied owing to their stable, highly tunable, and efficient band-edge emission and controllable charge transport dynamics. CdSe-based optoelectronic devices have shown remarkable performance as the active material in LEDs,^9–13^ lasers,^14–16^ solar cells,^17,18^ luminescent solar concentrators (LSCs),^19^ field-effect transistors (FETs),^20,21^ and photodetectors.^22,23^

Generally, progress in photodetector devices has been made mainly utilizing diverse material systems,^24^ particularly focusing on device architecture,^25^ interfacial engineering and optimizing charge transport, to achieve high photoresponse performance.^26–30^ More recently, there has been considerable interest in exploring CdSe NPLs as the active absorbing material for photodetector applications because of their exceptional properties, including large optical absorption cross-section, adjustable optical bandgap, and high charge mobility.^31,32^ Nevertheless, the performance of these NPL-based photodetectors still lags behind that of the other classes of materials, mainly due to the photocarrier generation and complex transportation management.^33,34^ Several strategies have been utilized to overcome these challenges, including charge doping with Mn^2+^,^35^ Cu^2+^,^36^ Eu^3+^,^37^ and In^3+^,^38^ integration with graphene^39,40^ and MoS_2_,^41^ and adopting complex heterostructures.^42,43^

Self-assembly of nanocrystals into an ordered configuration is yet another powerful and cost-effective strategy that has been utilized recently to facilitate charge transportation.^44,45^ This solution-processed deposition technique enables efficient electronic coupling between the adjacent nanocrystals, which ultimately provides the shortest path for charge carriers to travel from one electrode to the other.^46,47^ To further enhance the charge carrier mobility, the long and bulky organic ligands of the as-synthesized nanocrystals, which impede the charge transport within the assembled nanocrystals,^48^ must be exchanged with shorter ones. The ligand exchange is vital for improving the role of self-assembly in charge transportation and decreasing the hopping distance.^49^ Several attempts have been made to improve the performance of optoelectronic devices utilizing oriented nanocrystals. Shi et al.^23^ reported light-induced assembly under an external electric field to enhance the photoresponse of the CdSe nanowire photodetector. The impact of aligned assemblies of CdSe/CdS core/shell nanorods on photoconductivity by drop-casting on a prepatterned substrate utilizing the coffee stain effect was investigated by Persano et al.^50^ Recently, our previous work demonstrated the profound impact of liquid–air self-assembly of CdSe NPLs on photodetection performance in terms of detection speed, responsivity, and detectivity utilizing planar-configuration metal–semiconductor-metal (MSM) interdigitated electrodes.^22^

With all these efforts, however, the photodetection performance of oriented NPLs in vertical configuration photodetector devices has not yet been explored, although vertically assembled NPLs are theoretically expected to yield enhanced photodetection performance in the vertical configuration due to the short charge transportation distance and high vertical electrical fields acting on the photogenerated charges to separate and collect them across the vertical device. Yet, the key question is whether the performance advancement associated with the uniform orientation nanoplatelets assembly, already reported in planar configuration photodetectors, remains relevant at significantly shorter transportation length, as is the case in vertical configuration photodetectors, where charges have to travel one or a few nanoplatelets. This is a critical issue that cannot be addressed by the planar device configuration alone.

Herein, we experimentally studied the photocurrent response of self-assembled CdSe NPLs systematically arranged in edge-up and face-down orientations, providing valuable insights compared to randomly oriented NPLs. The charge-transport and relaxation dynamics were shown to be very sensitive to the configuration of the NPLs in the vertical device architecture. The carrier pathway was minimized by assembling the NPLs to provide the shortest route and minimizing the hopping instants between the collecting electrodes by utilizing a vertical photodetector device structure with ultrathin active material. Moreover, the original bulky ligands of the NPLs were exchanged with short ones, which further facilitated the carrier mobility and nullified the possible trap sources on the NPL’s surface. The performance of the vertical photodetector devices was investigated in terms of photocurrent responsivity and detectivity, showing highly promising potential for the next-generation, atomically thin optoelectronics and sensors. The innovative device fabrication approach presented here possibly paves the way for exciting advancements in colloidal security and fast image sensors.

## 2 Materials and Methods

### 2.1 Synthesis of 4 ML CdSe Nanoplatelets (NPLs)

Four monolayers (MLs) CdSe core NPLs were synthesized according to a recipe in the literature.^51^ In a 100 mL 3-neck flask, 340 mg of cadmium myristate, 24 mg of Se, and 30 mL of ODE were mixed and degassed at 95 °C for 1 h to remove oxygen and other volatile species. Then, the flask was flushed with Ar gas, and the temperature was set to 237.5 °C. At 195 °C, 120 mg of Cd­(OAc)_2_ × 2H_2_O was swiftly added to the flask. The solution was kept at 237.5 °C for 8 min, and then 1 mL of OA was injected into the solution, after which the flask was quenched in a water bath. The solution was collected, diluted with 6 mL of hexane, and centrifuged at 6000 rpm for 6 min to remove the unstable NPLs. Through the addition of extra ethanol to the supernatant and centrifugation, 4 ML CdSe core NPLs were precipitated and redispersed in hexane.^52^

### 2.2 Ligand Exchange of CdSe NPLs with EHT

Under Ar flow, 150 μL of 2-ethylhexane-1-thiol (EHT) was added to 1 mL of OA-capped NPLs in toluene (100 mg/mL). The solution was stirred for 2 h, then the NPLs were precipitated by adding ethanol and redispersed in toluene. The process was repeated twice with 50 μL of EHT in the second and third cycles for a complete ligand exchange. Finally, the resultant NPLs were washed with ethanol and redispersed in hexane.^9^

### 2.3 Device Fabrication and Characterization

Prepatterned indium tin oxide (ITO) substrates (purchased from Osilla) with dimensions of 20 × 15 mm^2^ were thoroughly cleaned using Hellmanex detergent, distilled water, acetone, and isopropanol with 10 min sonication and then dried with airflow. Subsequently, CdSe NPL layers were deposited on the substrates utilizing two different deposition techniques: spin-coating, yielding random orientations of the CdSe NPL, and liquid–air self-assembly, enabling either the desired all face-down or all edge-up orientation of the NPLs, depending on the self-assembly conditions. For the spin-coating film, a 10 μL single drop (25 mg/mL in hexane) was spin-coated at 2000 rpm for 60 s in the air, and for the self-assembly deposition of face-down and edge-up monolayer films, 10 and 20 μL single drops with concentrations of 25 mg/mL in hexane and 20 mg/mL in octane were used, respectively. The used concentrations and solvents were thoroughly optimized to ensure a homogeneous spread of the NPLs on the subphase solvent to result in a well-packed monolayer of the assembled NPLs. The deposited films were baked at 120 °C for 30 min in the glovebox filled with nitrogen (O_2_ < 0.1 and H_2_O < 0.1 ppm) to ensure the evaporation of the remaining solvent. Finally, the Al backside electrode with 100 nm thickness was deposited directly on the CdSe NPLs using a shadow mask with an active area of 4.5 mm^2^ in an oxygen-free thermal evaporator system under a pressure level of 7.5 × 10^–7^ Torr.

The photoconductivity and temporal response of the photodetector devices were characterized with a probe station using a computerized semiconductor analyzer (Agilent B1500A). A blue LED at a wavelength of 455 nm using variable light intensities (Thorlabs), calibrated using a Si photodiode from Newport, has been used as the excitation light source. A functional generator has optimized the light cycle period to 0.5 Hz. The absorption and photoluminescence spectra of CdSe NPLs films of different orientations deposited on a quartz substrate were collected using an Agilent Cary 60 UV–vis Spectrophotometer and a Varian Cary Eclipse at an excitation wavelength of 400 nm, respectively.

### 2.4 Absorbance Spectra Processing

We followed the procedure below to remove the scattering background from absorbance spectra. Scattering of light scales in a power law with wavelength: σ­(λ)∝λ^–m^. The value of m changes from 4 to 2 as the area of 2D nanostructures increases, which confirms a transition from Rayleigh to van der Hulst scattering.^53^ In our case, we subtracted C × λ^–m^ from our absorbance curves until we obtain a constant level of absorbance that closely matches the absorbance spectrum of CdSe NPLs in solution, which is nearly zero for the wavelengths longer than the heavy hole transition (550 nm<λ< 800 nm). C is an adjustable constant number for each fitting. This is required since our CdSe NPLs are optically transparent for 550 nm<λ<900 nm, meaning that the absorbance is nearly zero in this range.

For *m* > 2, we observed that we cannot obtain a constant near-zero absorbance after removing the calculated scattering for the wavelengths longer than the wavelength of the heavy hole transition (550 nm<λ< 800 nm). For *m* = 2 after the removal of the scattering part, we obtained the best results, meaning that we obtain a constant absorbance, which is nearly zero for 550 nm<λ< 800 nm, and for λ<550 nm we obtain an absorbance spectrum that closely matches the absorbance spectrum of CdSe NPLs in solution.

## 3 Results and Discussion

Four monolayer (ML) CdSe NPLs with lateral dimensions of 13 × 13 nm^2^ and a thickness of 1.2 nm were synthesized according to a recipe developed by our group with slight modifications.^51,54,52^ The NPLs were originally passivated with native oleic acid (OA) organic ligands for their colloidal stability. However, the long and bulky organic chain of OA heavily undermines charge transport by increasing the interparticle spacing within the assembled NPLs. Herein, OA was replaced with short-chain 2-ethylhexane-1-thiol (EHT) ligands to reduce the interparticle distance and improve the electronic coupling between the adjacent NPLs.

The transmission electron microscopy (TEM) images of the NPLs capped with OA and EHT are shown in Figures 1a,b, respectively. The NPLs retained their 2D shape and size after ligand exchange with partial assembly and chain formation on the TEM grid. The Fourier-transform infrared spectroscopy (FTIR) spectra of CdSe NPLs before and after the ligand exchange reaction, shown in Figure 1c, confirm the effective ligand exchange process. The emergence of the absorption peak at 669 cm^–1^ associated with the C–S bond of the EHT ligands and the complete removal of the prominent C=O stretch peak at 1710 cm^–1^ and C=C stretch peak at 1650 cm^–1^ provide strong evidence for the OA ligands removal and were completely exchanged with EHT.^55,56^

**Figure 1:**
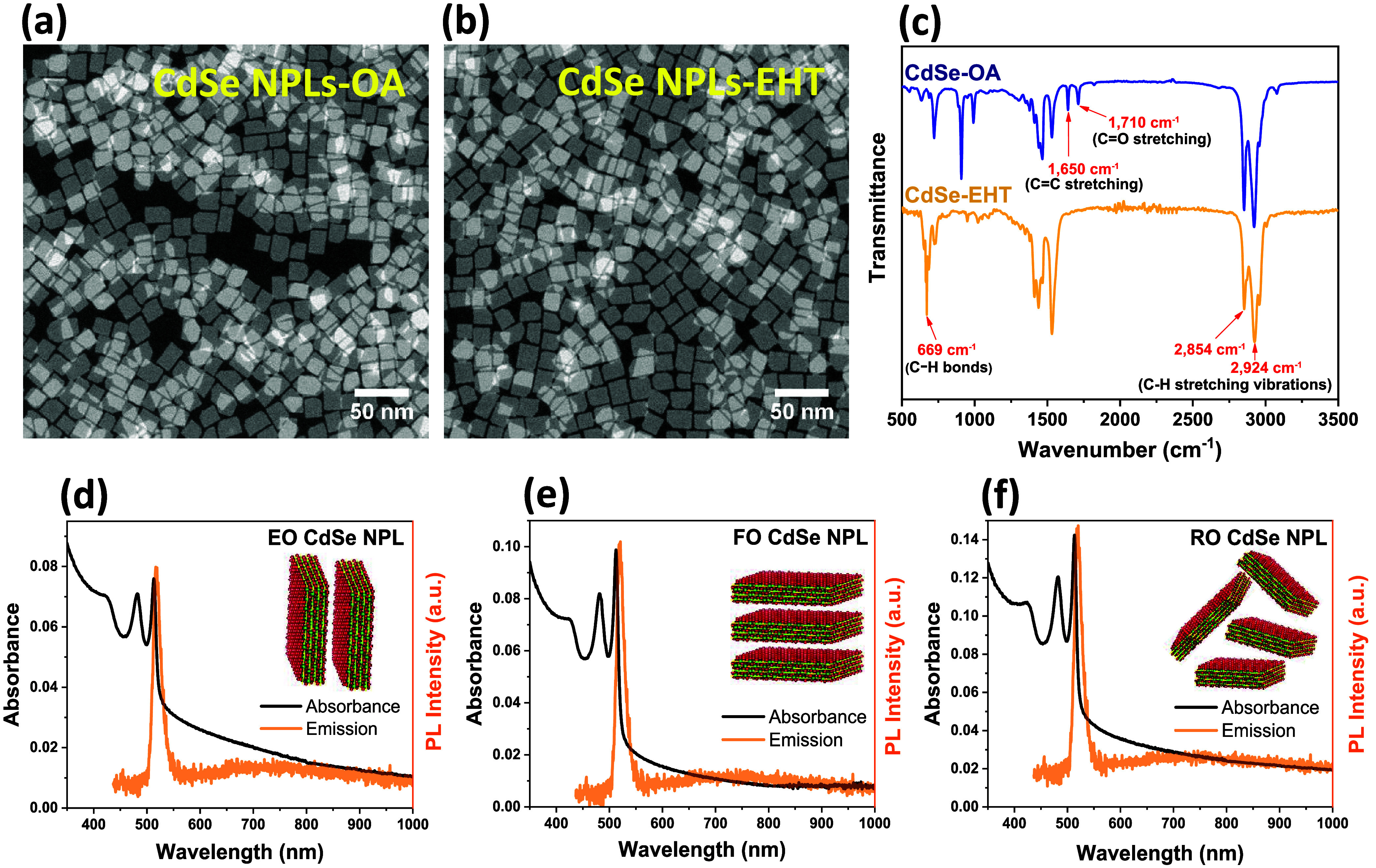
Characterization of CdSe NPLs. TEM images of (a) as-synthesized NPLs and (b) NPLs after ligand exchange with EHT. (c) FTIR spectra of NPLs with OA and after ligand exchange with EHT. (d–f) Absorbance and PL spectra of NPL films of edge-up orientation (1EO), 3 layers of face-down orientation (3FO), and random orientation (RO), respectively, deposited on a quartz substrate.

Charge-transport dynamics and pathways can best be investigated by controlling the NPL’s orientation. Such unique control can be achieved by utilizing the liquid–air interface self-assembly technique, adapted and demonstrated for NPLs by our group,^22,54,9,57,58^ where the NPLs’ orientation can selectively be controlled to be either all face-down (lying on their sizable lateral face) or all edge-up (closely standing together by their edges). We utilized this self-assembly technique to study the photoresponse of uniformly oriented NPLs in EO and FO assemblies as opposed to RO film deposited by spin-coating within a vertical photodetector device architecture.

Figure 1d–f show NPL films’ absorption and photoluminescence (PL) spectra on quartz substrates with 1EO, 3FO, and RO assemblies, respectively. All films feature the characteristic excitonic absorbance peaks of CdSe NPLs with slight differences in their intensities due to film thickness and dielectric constant variations. The PL spectra of the films with different NPL orientations show a narrow emission peak centered at 513 nm with a full-width at half-maximum (FWHM) of ∼10 nm. The recorded optical response of the CdSe NPL films shows minimal dependence on the NPL orientation, a clear indication that the assembly configuration has no tangible effect on the exciton generation and dynamics. Therefore, we can primarily attribute pronounced orientation-dependent differences in the photodetectors’ performance, discussed later, to charge transportation and the interfacial band alignment with the electrodes.

Vertical configuration photodetector devices are well-known for their fast response to optical signals due to the strong effect of the electric field on the photoactive semiconductor, which leads to efficient separation of the electron–hole carriers.^59^ The ability to selectively adjust the CdSe NPLs orientations between the top and bottom metal electrodes provides a unique tool to shorten the semiconductor’s photoresponse time by controlling the photogenerated carriers’ path length. Furthermore, the efficient passivation of the NPLs surface with the short EHT ligands, as confirmed by the FTIR shown in Figure 1c, diminishes the charge trap centers that undermine the charge carrier’s density and recovery time and creates a shorter transportation pathway to the electrodes.

In the 1EO device, a single layer of the CdSe NPLs has been directly used as the active material of the photodetector device, which is found to be sufficient for a stable working device. In contrast, for the FO device, 1 to 2 monolayers were insufficient to prevent short-circuiting problems (Figure S1). We attribute this to the intrinsic differences in CdSe NPL arrangement within the film formation between the two assemblies. The failure of the 1 and 2 ML FO films to make a working device strongly suggests that ligand packing on the (100) edge facets of the CdSe NPLs is insufficient to prevent the diffusion of the Al atoms during the thermal evaporation process. However, in EO film, in addition to the height-to-gap ratio of the CdSe NPLs, the NPLs’ basal plane-to-basal plane coupling prevents the device from short-circuiting. To overcome this problem, we fabricated the FO device with three consecutive NPL layers (3FO). Having a third NPL layer in the FO device not only eliminates the short-circuiting problem but also provides a film thickness comparable to that of the 1EO device, facilitating a meaningful comparison in device performance.

The final 3FO film demonstrates high uniformity, with no cracks or voids (SEM in Figure S2). The SEM images of 1EO, 3FO, and RO films are shown in Figure 2a–c, respectively, with large-scale SEM images in Figure S3, confirming the films’ high uniformity and long-range coverage. The electron transport path within these assembled orientations is schematically depicted in Figure 2, where directional routes in self-assembled NPL films result in a straight pathway. As mentioned earlier, the vertical architecture device ensures better charge transportation due to the decreased number of hopping steps, which, in an ideal case, implies only 1 or 2 hopping steps from one electrode to the other electrode. In contrast, the electron transportation routes in randomly oriented NPL films (RO) are drawn in curved arrows to emphasize the longer pathway of electrons. The indirect pathway consists of more NPL involved in electron transportation and increased hopping incidents before the electrons are collected, leading to a longer electron transportation route.

**Figure 2:**
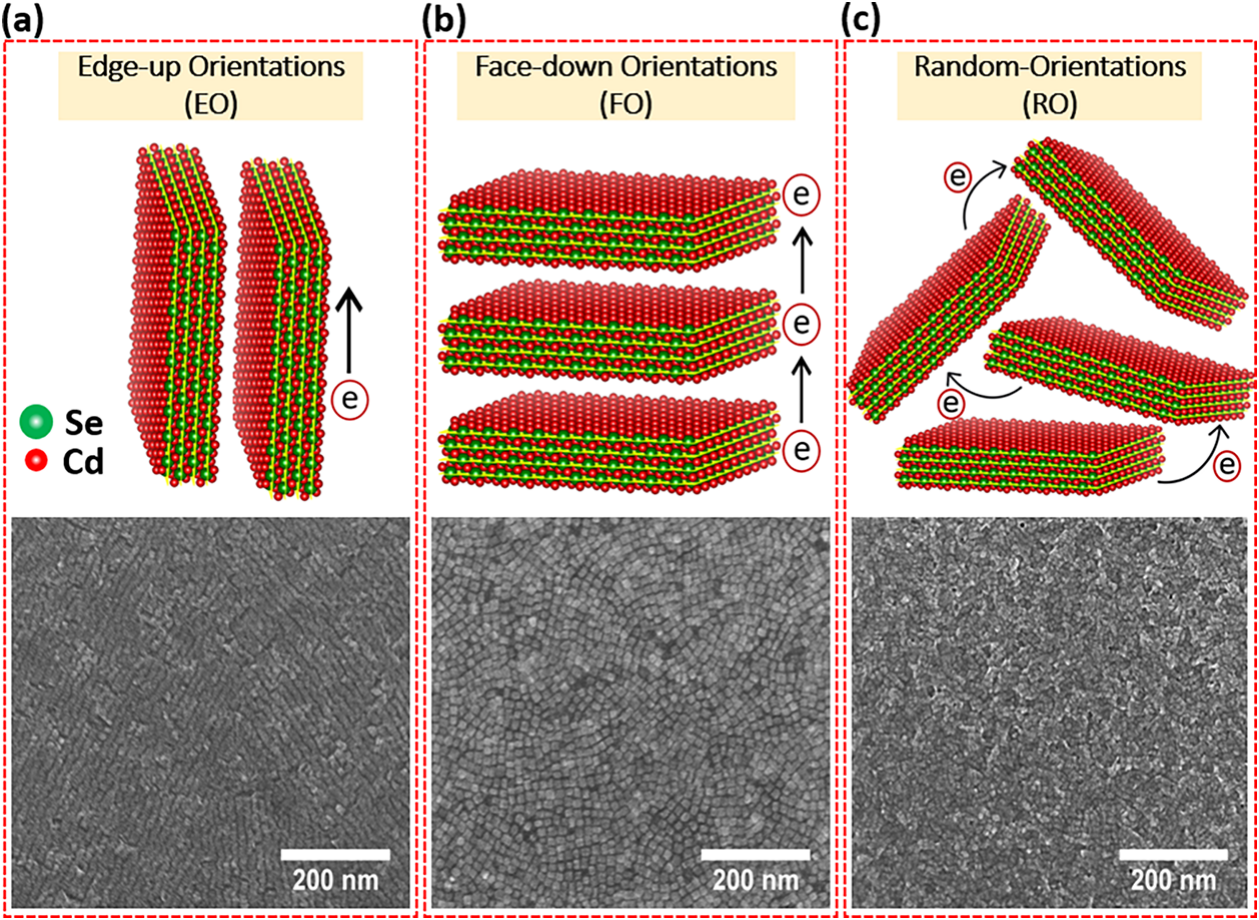
Schematic illustrations of possible charge transportation pathways within the CdSe NPL-based photodetector devices using various NPL orientations with their corresponding SEM images: (a) 1EO, (b) 3FO, and (c) RO, respectively.

The photodetector device in vertical configuration utilized in this work is schematically illustrated in Figure 3a, with the inset showing the energy level alignment of the CdSe NPLs with respect to the contact electrodes. The NPL film was deposited with the desired orientation on a precleaned patterned ITO substrate, and then the back Al electrode was deposited through a thermal evaporation system at 6 × 10^–7^ Torr. Each device substrate contains 8 identical photodetector devices fabricated under the same preparation conditions, all of which show consistent photodetection performance, a clear indication of good device stability and structure over the time scale of fabrication and measurement. The cross-sectional SEM image of the photodetector device with randomly oriented NPLs in Figure 3b shows the formation of a successive and smoothly separated photoactive layer and contact electrodes.

**Figure 3:**
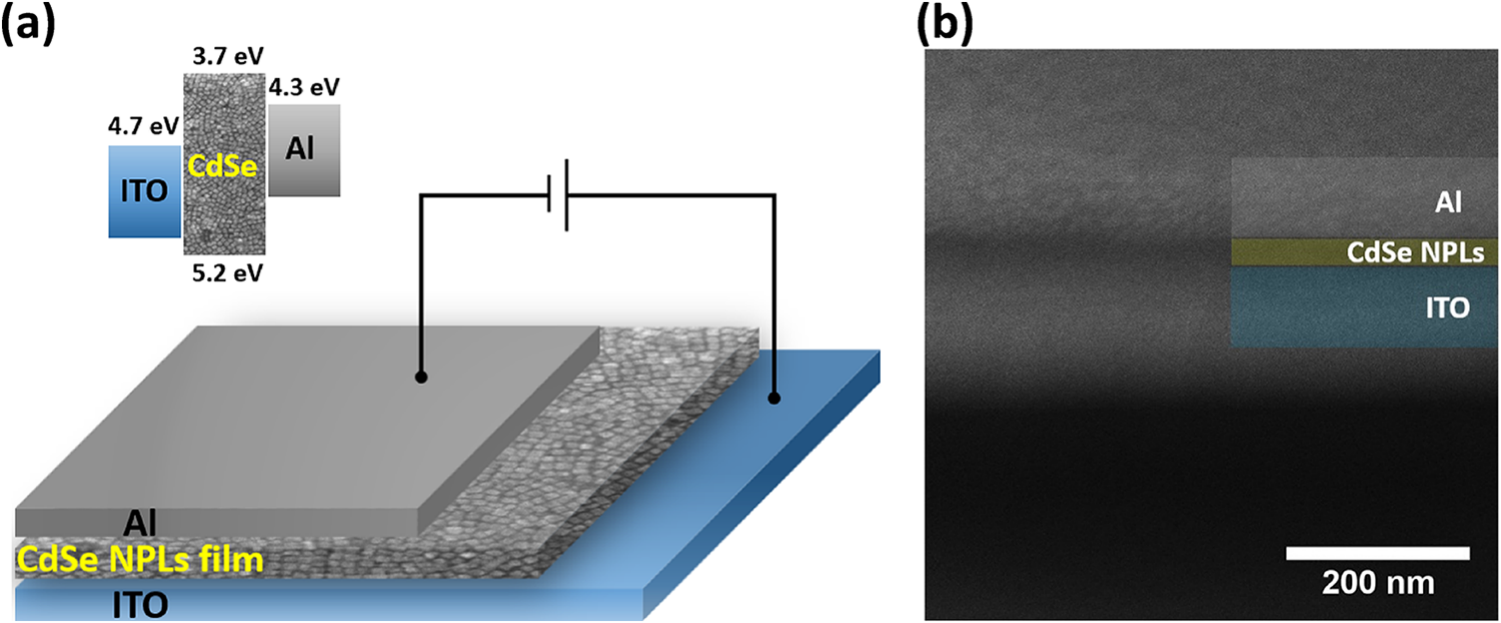
(a) Schematic illustration of the photodetector device with vertical architecture. The inset shows the energy band alignment of CdSe NPLs with respect to the contact electrodes. The interface energy values were adopted from published literature,^22,60,61^ (b) Cross-sectional SEM image of the device with a partially false-colored region to indicate the respective layers. The scale bar is 200 nm.

Figure 4a shows semilog *I*–*V* characteristic curves in the dark (dots) and under illumination (line) using a power density of 63.42 mW/cm at 455 nm for the devices with 1EO, 3FO, and RO films. The asymmetric *I*–*V* characteristics show a monotonic increase in photocurrent with increasing voltage under light irradiation, in contrast to the dark mode. However, the photodetector device with RO NPL exhibits higher photoconductivity than that of the 1EO and the 3FO orientations. Figure 4a is replotted on a linear scale, shown in Figure S4, to further clarify the asymmetric rectification behavior of the devices due to the different work functions of the contact metals.

**Figure 4:**
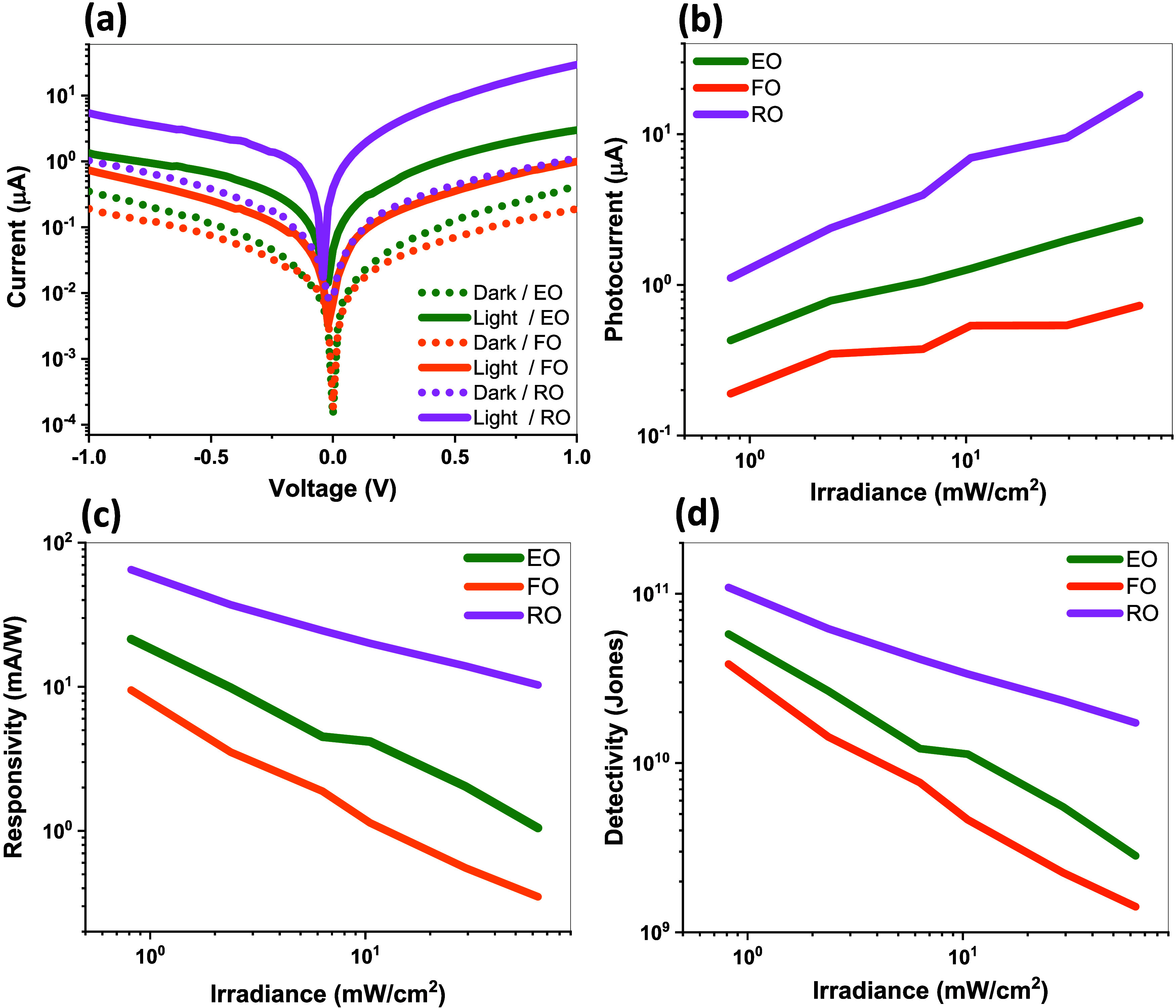
(a) Semilogarithmic *I*–*V* plot of the 1EO, 3FO, and RO NPL photodetector devices in dark mode and under LED illumination at 455 nm and 63.42 mW/cm^2^ power density. (b) Photocurrent vs. irradiance of the devices illuminated at 455 nm at a bias voltage of 1 V. (c) Log curves of the responsivity of the photodetector devices recorded at a bias voltage of 1 V. (d) Detectivity as a function of irradiance of the photodetector devices.

It should be noted that the NPL film thicknesses of the three devices differ due to variations in the deposition process. The RO film has a thickness of 22.5 nm, while 1EO and 3FO films have thicknesses of 13.5 and 12.7 nm, respectively, as measured by atomic force microscopy. Increasing the thickness of the NPL film enhances the photocurrent due to the elevated charge density. This effect is demonstrated in the photocurrent behavior of the devices illustrated in Figure 4a. The RO film shows photoconductivity of 29.4 μA, followed by the 1EO and 3FO films with 3 and 1 μA at a voltage bias of 1 V, respectively. Figure 4b shows the dependence of the photocurrent on irradiance at a fixed applied voltage of 1 V. The photocurrent increases monotonically with irradiance for all devices, indicating efficient charge separation and collection. The sublinear increment of the photocurrent with irradiance is attributed to the charge traps and/or recombination processes.^62^

It is important to realized that the absorbance measurements of the nanoplatelet films are strongly affected by light scattering within the films, which is a well-known phenomenon.^53^ The reported absorbance values of our films are comparable at the excitation wavelength of 455 nm and are largely affected by the light scattering. Therefore, the scattered background was removed from our absorbance spectra to obtain the actual absorbance of the films and the percentage of the absorbed incident photons, as shown in Figure S5.

According to the normalized spectra, the absorbance of 1EO, 3FO, and RO is 0.015, 0.031, and 0.035 at the excitation wavelength of 455 nm. Accordingly, 3.4% of the light is absorbed by the EO film, 7.1% of the light is absorbed by the FO, and 8% of the light is absorbed by the RO. Therefore, we can fairly state that the RO film is absorbing 2.34 times more photons than the EO film and 1.15 times more than the FO film. Since *n* ≪ 1 (*n* is the number of the generated excitons by our continuous-wave light source), the absorbance will be the same for the incident powers for the CdSe films. This means that the percentage of absorbed photons will remain the same for the incident powers. For example, the number of absorbed photons for the RO film at 1 mW/cm^2^ and the number of absorbed photons for the EO film at 2.34 mW/cm^2^ will be the same. To compare the device performances, we normalized the photocurrent and responsivity by the number of absorbed photons, as shown in Figure S6. In the normalized case, the photocurrents of EO and RO are 0.47 and 1.3 μA at 1 mW/cm^2^, where the photocurrent of RO is 2.7 times that of 1EO. While for the normalized case, the photocurrents of EO and RO are 0.8 and 1.3 μA at an irradiance of 1 mW/cm^2^, resulting in RO photocurrent being 1.6 times that of EO.

We further investigated the dependence of responsivity and detectivity on the irradiated light power density to evaluate the device’s performance. Figure 4c,d show the responsivity (*R*_λ_) and detectivity (*D* ^*^) of devices under illumination at 455 nm and a fixed bias voltage of 1 V, calculated by the following equations^22,63^

1 $$R_{\lambda }=\frac{I_{\mathrm{Ph}}}{P_{\lambda }\times A}$$

2 $$D^{{\;}^{*}}=\sqrt{\frac{A}{2eI_{\mathrm{dark}}}}\times R_{\lambda }$$

where *I*_Ph_ is the device photocurrent (*I*_Ph_ = (*I*_light_ – *I*_dark_)), *P*_λ_ is the light power density, *A* is the active area of the photodetector device, and *e* is the elementary charge. In our MSM vertical-configuration photodetectors, in the absence of illumination, the intrinsic noise is assumed to be the dominant contributor to the overall noise current signal, such as shot noise. The reported detectivity values represent an upper-bound estimation based on the shot-noise-limited dark current, which could therefore correspond to a theoretical limit rather than measured noise power spectral density. Therefore, the noise current for devices before and after ligand exchange (shown in Figure S7) was calculated from the measured dark current with a bandwidth of 1 Hz using the following equation^22,63^

3 $$i_{n}=\sqrt{2eI_{\mathrm{dark}}}$$

The photodetector devices show optical responsivity of 21.04, 9.05, and 65.00 mAW^–1^ for the 1EO, 3FO, and RO NPL devices, respectively, measured at the lowest incident power density of 0.8 mW/cm^2^. The same devices exhibit optical detectivity values of 5.77 × 10^10^, 3.84 × 10^10^, and 1.08 × 10^11^ Jones for the 1EO, 3FO, and RO NPLs, respectively. The responsivity and detectivity values reported for the self-assembled photodetectors are competitively high considering the atomically thin nature of the active CdSe NPL layer compared to CdSe-based photodetectors.^62,64^ For instance, Dutta et al.^65^ studied the photoconduction performance of spin-coated CdSe NPLs, showing responsivity and detectivity values of 0.007 mAW^–1^ and 3.7 × 10^9^ Jones, respectively. The performance of our assembled devices is especially interesting considering efficient optoelectronic devices with atomically thin active materials.

Figure 5 shows a semilog plot of the EQE performance versus applied voltage of vertical-configuration photodetectors made with CdSe NPLs of native Oleic acid and EHT ligands deposited at different nanocrystal orientations: 1EO, 3FO, and RO, calculated using the equation below^63^

4 $$\mathrm{EQE}\left(\%\right)=\frac{I_{\mathrm{ph}}\times h\times c}{P_{\mathrm{in}}\times e\times \lambda }\times 100\%$$

**Figure 5:**
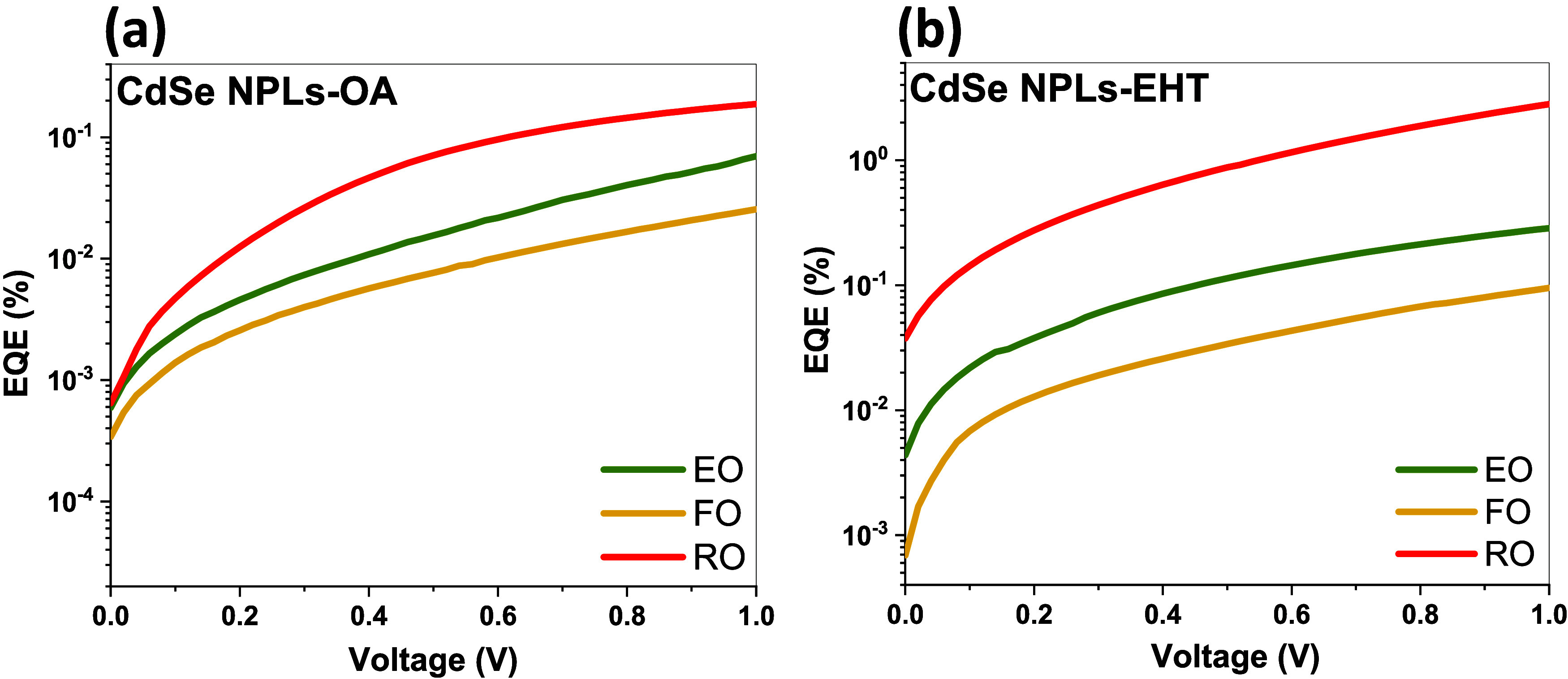
EQE of photodetector devices made of CdSe NPLs deposited at different nanocrystal orientations; 1EO, 3FO and RO, (a) CdSe NPLs with native Oleic acid ligands, (b) CdSe NPLs after ligands exchanged with EHT.

where *I*_ph_ is the photocurrent, *e* is the elementary charge, *P*_in_ is the incident optical power (power density (W/cm^2^)/device area (cm^2^)), *h* is Planck’s constant, *c* is the speed of light, and λ is the excitation wavelength (455 nm).

Figure 5a shows the EQE performance of the photodetectors of different CdSe NPL orientations with their native Oleic acid ligands. For the 1EO device, the EQE is 0.07%, which is lower than the RO device (0.18%) and higher than the 3FO (0.02%). Figure 5b shows the EQE performance after ligand exchange with EHT. The EQE of the 1EO device records a maximum value of 0.28%, an order of magnitude lower than the RO device, with a maximum EQE of 2.81% and approximately three times higher than the 3FO device, with EQE of 0.09%, all recorded at 1 V. EQE result shows that while edge-up oriented nanoplatelets is not as effective as the randomly oriented counterparts in maximizing the EQE efficiency, it surely indicates a more conductive pathway compared to the face-down orientation.

The temporal response of the photodetector device is a crucial performance aspect from a practical point of view, demonstrating the photoinduced charge carrier’s separation rate, reaching the collecting electrodes, and generating the electrical signal. The time-dependent photoresponse was recorded under LED light (63.42 mW/cm^2^ at 455 nm) modulated at 0.5 Hz at a fixed applied voltage of 1 V. All devices demonstrate high photocurrent switching stability and repeatability when illuminated, despite variations in their photoresponse time constants. Figure 6a–c show the temporal photoresponse of 1EO, 3FO, and RO NPL devices, respectively. All devices exhibit a time-dependent photoresponse, characterized by a spike-like increase in photocurrent upon light irradiation, followed by an exponential decrease in photoconductivity during illumination. This behavior has been observed previously^22,65^ and attributed to charge-trapping centers with varying numbers/concentrations in each device, consistent with the sublinear increase of photocurrent versus irradiance shown earlier in Figure 4b. The rise and decay photoresponse time constants are defined by the time duration needed for the photocurrent to increase from 10 to 90% of its maximum and vice versa, respectively. The rising transient photocurrent of the uniformly oriented devices of 1EO and 3FO (both with OA and EHT) can best be described by a monoexponential fit component (eq 5), as illustrated by the fitting red lines in Figures 6a,b and S8a,b. While a double-component exponential function (eq 6) is needed to fit the rising time of the RO device with OA ligand.

5 $$I\left(t\right)=I_{o}+A\exp\left(\frac{t}{\tau_{1}}\right)$$

6 $$I\left(t\right)=I_{o}+A_{1}\exp\left(\frac{t}{\tau_{1}}\right)+A_{2}\exp\left(\frac{t}{\tau_{2}}\right)$$

**Figure 6:**
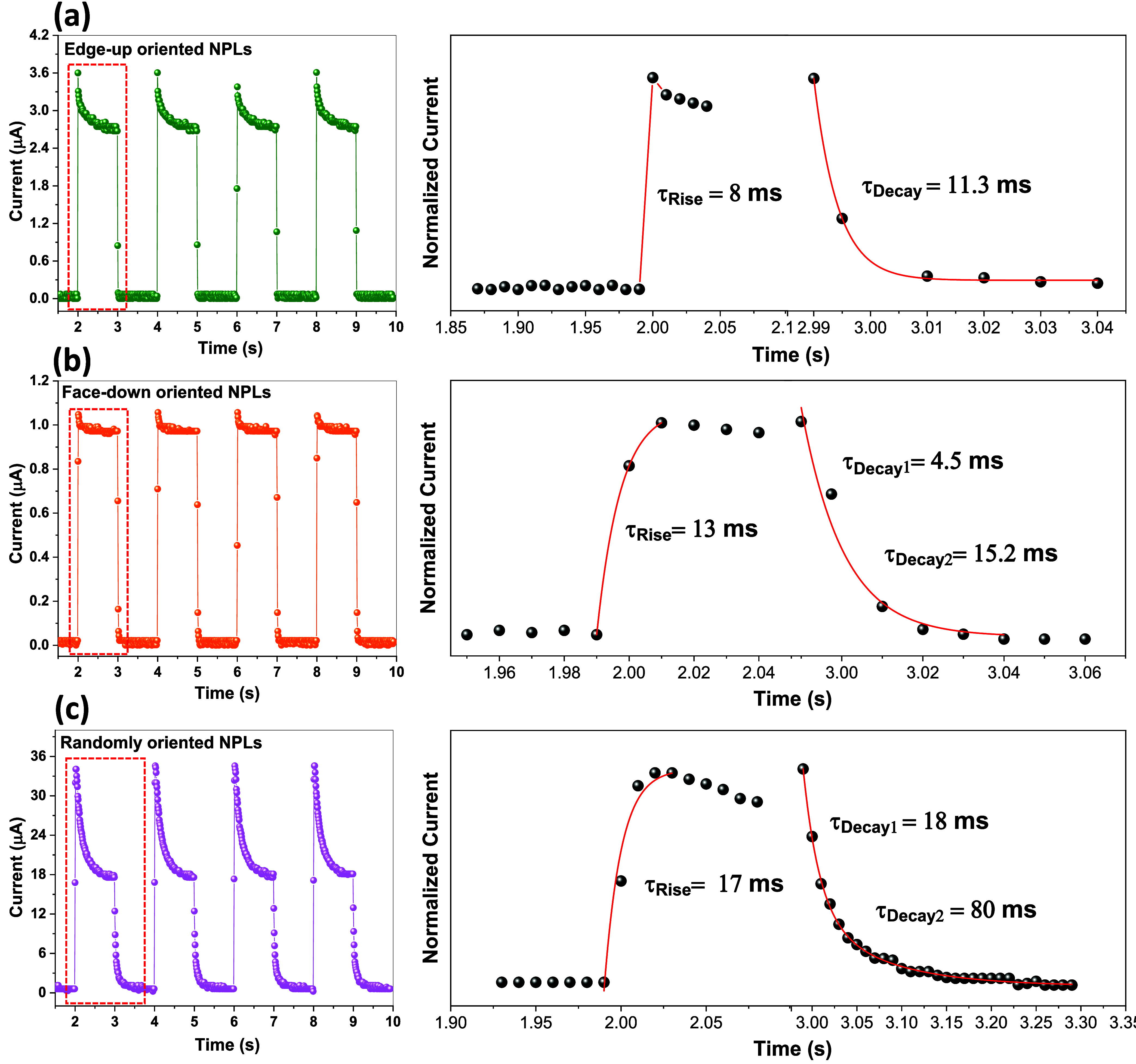
Temporal photoresponse and their enlarged corresponding fitting of the photodetector devices using various NPL orientations: (a) 1EO, (b) 3FO, and (c) RO, all under a bias voltage of 1 V. The devices were excited with a 455 nm LED at 0.5 Hz optical cycle modulation.

Where *I*(*t*) is the measured current as a function of time (t), *I*_o_ is the baseline current measured at *t* = 0, *A* is the photocurrent amplitude, and τ_1_ and τ_2_ representing the rising time constant components. On the other hand, the decay time constant of the 1EO device of CdSe NPLs with EHT ligands (Figure 6a) is fitted with a monoexponential fit (eq 7), unlike all other devices, where their decay transient photocurrents were fitted with a double exponential function (eq 8).

7 $$I\left(t\right)=I_{o}+A\exp\left(\frac{-t}{\tau_{1}}\right)$$

8 $$I\left(t\right)=I_{o}+A_{1}\exp\left(\frac{-t}{\tau_{1}}\right)+A_{2}\exp\left(\frac{-t}{\tau_{2}}\right)$$

Charge transport pathways and trap-assisted recombination dynamics were studied based on the CdSe NPLs orientation by fitting the rise and decay portions of the transient photocurrent. Table 1 compares the temporal photoresponse time constants of the studied CdSe NPL photodetector devices with native OA ligand and after ligands have been exchanged with EHT.

**Table 1:** A Comparison of the Transient Photocurrent Time Constants of Photodetector Devices of Different CdSe NPL Orientations with Native OA Ligands and after Being Exchanged with EHT Ligands

	time constants (ms)			
	rise time		decay time	
nanoplatelets orientation	τ_1_	τ_2_	τ_1_	τ_2_
1EO-OA	10	--	6	32
3FO-OA	16	--	17	57
RO-OA	46	55	54	110
1EO-EHT	8	--	11.3	--
3FO-EHT	13	--	4.5	15.2
RO-EHT	17	--	18	80

The electrical performance observed in our devices indicates the complexity of the interfacial energetics associated with the fundamentally different crystallographic facets of the NPLs interfacing with the Al back electrode. These crystallographic variations will impose different surface energies and ligand packing densities of the nanoplatelet facets, leading to distinct interface dipoles for the specific NPL arrangement when interfaced with the metal electrode.^66,67^ A band diagram illustrating the energy alignment of CdSe NPL corresponding to the contact electrodes in 1EO, 3FO, and RO devices is schematically shown in Figure 7. The figure depicts the orientation-dependent electronic structure of different facets when interfaced with the Al electrode. The energy levels are depicted as a flat band in Figure 7 for simplicity. The potential barrier at the interfaces to the NPL film in the structure is likely a continuous gradient (strong internal field) rather than a discrete interfacial step.^68,69^ In the EO films, the NPLs edges with high density of defects are close to the electrode, enabling a high density of trap states and a low interfacial potential barrier (Figure 7a). These trap-rich sites have the nature of shallow donor/acceptor-like states that facilitate hopping-like charge transport within the neighboring NPLs that outpaces the nonradiative recombination processes. However, in FO films, because these trap-rich sites (narrow facets) are relatively far from the electrodes, the density of trap states is lower than in EO, thereby enabling a higher energy barrier, as depicted in Figure 7b. In the case of RO films, a mixture of NPL orientation will be interfacing with the electrode, leading to intermediate energy trapping states density and barrier compared to EO and RO, as shown in Figure 7c.

**Figure 7:**
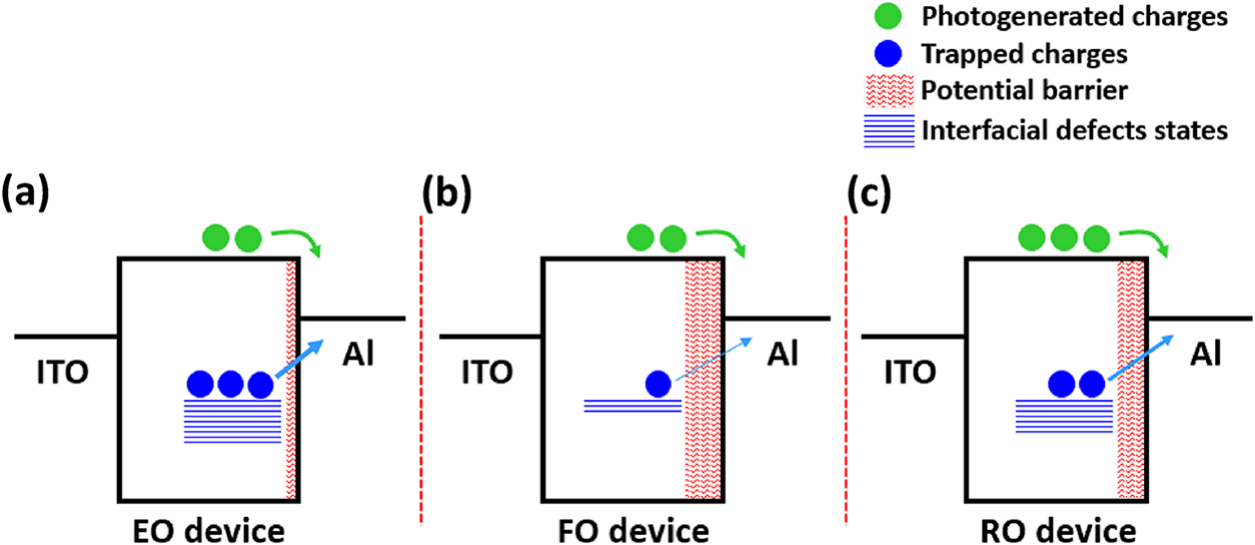
Energy band schematic illustration of the nanoplatelets orientation-dependent charge injection governed by the orientation-dependent electronic structure at the interface with the Al electrode for photodetector devices of (a) 1EO, (b) 3FO, and (c) RO.

Additionally, our devices also show variation in photocharge transportation pathways enabled by the nanoplatelets’ self-assembly deposition. Therefore, these critical factors will be considered when discussing the photodetector device performance.

In the 1EO device, the contact metal electrodes primarily interface with the spatially narrow, 100 edge facets of the CdSe NPLs, with a high density of uncoordinated atoms and low ligand coverage, thereby enabling strong electronic coupling with the electrodes. This contact arrangement results in fast charge extraction dynamics. This is consistent with the fast photocurrent transient time constants observed in the 1EO device. Furthermore, in the 1EO device geometry, the photocharge carriers must traverse along the nanoplatelet lateral surface to be collected by the electrodes. Therefore, the faster response in the 1EO device is attributed to both the reduced hopping pathways and the crystallographic orientation facilitated by the self-assembly deposition technique. This means that no internanoplatelet hopping incident will be involved, and charges are expected to only move within the single crystalline NPL. As a result, the temporal responses of the 1EO devices with the OA ligand exhibit a fast rise time and a relatively simple current decay dynamics dominated by short and long-time components resulting from efficient carrier extraction or recombination and limited trap-assisted recombination, respectively (Figure S8a). After ligand exchange with EHT, the 1EO device shows even faster rising times and a single-exponential decay component (Figure 6a), a strong indication of enhanced electronic coupling at the NPL-electrode interface and efficient surface traps passivation.

In contrast, the metal electrodes in the 3FO devices primarily contact the lateral surface with 001 facet planes of the CdSe NPL, which are strongly passivated with ligands, resulting in partial electronic decoupling with the metal electrodes. This contact arrangement results in a significantly stronger surface dipole with the Al electrode compared to the 1EO device, which impacts the transport processes. Such a configuration affects the charge extraction processes, despite nanocrystal uniformity, leading to slower biexponential decay dynamics compared to the 1EO device. In terms of charge transportation, the 3FO device has three subsequent stacked layers of CdSe NPLs in a mosaic-like arrangement sandwiched between the electrodes. Charges have to hop across multiple NPL interfaces (energetic barriers) in order to be extracted by the electrode. Although the overall film thickness is equivalent to the 1EO device (about 13.5 nm), the sequential hopping process leads to a slower rising time and a biexponential decay behavior reflected by the existence of carrier trapping at the internanoplatelet interfaces (Figure S8b). The devices with EHT ligands exhibit improved performance in terms of rising and decay times, where the latter show a reduction in both decay time components due to efficient inter-NPL electronic coupling (Figure 6b). However, the current decay still needs a biexponential fitting function, a clear indication of the hopping transport limiting factor.

In the RO device, the metal electrodes contact a mixture of facet planes from a thicker (22.5 nm) and disordered nanoplatelet film, resulting in spatially inhomogeneous interfacial contacts across the metal–semiconductor region, which reduces charge carrier mobility through trap-assisted recombination processes. Charges in the RO device are expected to transport through a complex percolation pathway of lateral and vertical hopping incidents. Such a transportation movement imposes a significant reduction in the rise and decay time constants due to the high density of charge traps. The EHT ligands exchange process shortened the time constants, but trap-assisted recombination is still dominant due to structural disorder and a long, nonuniform transportation pathway. This explains the persistent photocurrent transient observed in the RO devices, as shown in Figure 6c.

Overall, it is clear that the photoresponse dynamics in the solution-processed vertical configuration photodetector device are governed not only by the interfacial electronic structure variation with the metal electrodes at distinct CdSe NPL facets but also by the transportation distance of charge carriers.

To provide a qualitative comparison of charge transport dynamics within the three devices, a rough estimation of carrier mobility (μ) is calculated based on the transient time approximation approach based on their rise time component, using the following equation^70^

9 $$\tau_{\mathrm{tr}}\approx \frac{L^{2}}{V\mu }$$

where *L* is the charge transport pathway, the film thickness in this case, *V* is the applied bias, and τ is the transient time constant (rise or decay time). However, this estimation approach does not take into consideration many effective factors which directly contribute to charge transport in such systems, such as trapping and detrapping, recombination and interfacial barriers. Therefore, the mobility data calculated for each device based on the above equation only describes an approximate effective mobility, which can only be used for a qualitative comparison. We have estimated the effective mobility in the three devices based on the transient time approximation using their rise time component and found it tobe μ_EO_ ≈ 2.28 × 10^–10^cm^2^V^–1^s^–1^, μ_FO_ ≈ 1.40 × 10^–10^cm^2^V^–1^s^–1^ and μ_RO_ ≈ 2.98 × 10^–10^cm^2^V^–1^s^–1^.

The temporal response in our assembled photodetector devices is several times faster than that reported in the literature for hybrid and CdSe-based photodetector devices, summarized in Table 2. Despite the promising temporal response characteristics of our devices, further device optimization would enable higher performance metrics comparable to state-of-the-art hybrid photodetectors. Furthermore, our results show a trade-off between photoresponse speed and sensitivity-related metrics in the vertical photodetector devices with nanoplatelet orientation. 1EO NPLs in vertical configuration photodetector devices reduce carrier transit time and trapping effects, providing rapid photoresponse performance. In contrast, RO devices show significantly higher photocurrent, responsivity, and detectivity due to its thicker film thickness, leading to higher optical absorption and photocarrier generation at the expense of sensitivity speed.

**Table 2:** A Comparison of the Photodetection Performance of CdSe-Based Photodetectors of Various Associated Material Systems and Different Configurations Reported in the Literature and This Work^a^

		photoresponse time (ms)				
device structure	bias (V)	rise	decay	*R*_λ_ (mA/W)	D* (Jones)	refs
CdSe NPLs	2	185	250	0.007	7.3 × 10^9^	^65^
CdSe NPLs	2	400	420	-	-	^71^
CdSe NPLs-Au NCs		200	240	18	2.4 × 10^11^	
CdSe NPLs	1.5	300	335	3.2	9 × 10^8^	^72^
CdSe NPLs-PTZ hybrid		107	110	70	4 × 10^11^	
CdSe QDs-ZnO	0	17.9	18	10.23	8.8 × 10^9^	^73^
MoS_2_/CdSe hybrid	0.5	60	60	2.5 × 10^10^	2 × 10^14^	^74^
Aligned CdSe NW film	1	20	40	-	-	^23^
CdSe NPLs (edge-up)	5	48	60	94.2	2.3 × 10^11^	^22^
CdSe NPLs (face-down)		61	74	6.17	1.7 × 10^10^	
CdSe NPLs (random)		100	160	528.7	7.5 × 10^11^	
CdSe NPLs (edge-up) (13 nm)	1	8	11.3	21.04	5.77 × 10^10^	this work
CdSe NPLs (face-down) (12.7 nm)		13	4.5, 15.2	9.05	3.84 × 10^10^	
CdSe NPLs (random) (22.5 nm)		17	18, 80	65.00	1.08 × 10^11^	

a The active layer film thickness of the devices investigated in this work was added to the device structure.

## 4 Conclusion

In summary, we successfully demonstrate photoresponse enhancement of NPL photodetectors in vertical configuration by assembling 1 and 3 monolayers of NPLs in EO or FO films utilizing the liquid–air self-assembly technique. The high uniformity of the assembled NPL films resulted in reduced charge trapping and accelerated charge carriers’ extraction by shortening the transportation pathway between the collecting electrodes. The 1EO and 3FO NPL photodetectors demonstrate extra-fast rise/decay time constants of 8 ms/11.3, 13 ms/4.5, and 15.2 ms, respectively, compared to 17 ms/18 and 80 ms for the spin-coated RO NPL device. Despite using one monolayer of NPLs in the EO and three monolayers in the FO films, the devices show highly promising performance with responsivity of 21.04 and 9.05 mAW^–1^ and detectivity of 5.77 × 10^10^ and 3.84 × 10^10^ Jones, for the 1EO and 3FO, respectively, with no compromise in performance compared to relatively thick CdSe-based photodetectors in the literature. We believe that the superior photoresponse speed of the 1EO device results mainly from the efficient interfacial electronic contact between the NPL facet and the Al electrode, and also from the uniform and short charge pathways provided by the assembly deposition processes that have been facilitated by the better film integrity. The results also reveal a clear trade-off between temporal response speed and sensitivity-related metrics as a function of the nanoplatelet orientation in vertical photodetectors. The findings of this study further emphasize the importance of nanocrystal arrangement for optoelectronic device performance and provide a practical approach for preparing high-performance ultrathin NPL photodetectors for the next-generation optical sensing. We distinctly find that 1EO and 3FO devices exhibit improved photoresponse and are therefore well-suited for applications that require atomically thin active materials with fast detection speed, such as high-speed sensing and optical communication. Conversely, applications where high responsivity and detectivity are prioritized over detection speed are better suited to RO devices. This orientation-dependent trade-off indicates the importance of structural control in optimizing vertical photodetectors for specific application domains.

## Supplementary Material



## Notes

I–*V* characteristics of the devices, SEM image of films, absorbance of the films, normalized device characteristics, and temporal photoresponse of devices fabricated using NPLs with OA ligands (PDF)
