# Supplementary material for: Edge-Up Oriented Colloidal CdSe Nanoplatelets Facilitate Faster Response in Vertical Photodetectors
Source: ACS Appl Mater Interfaces. 2026 Jun 29;18(27):37854–66. doi: 10.1021/acsami.6c04330 (PMC13383279; doi:10.1021/acsami.6c04330)
Supplement: Supplementary file 1 [file am6c04330_si_001.pdf]

Supporting Information

Edge-Up Oriented Colloidal CdSe Nanoplatelets Facilitate Faster Response in Vertical Photodetectors

Mohammed A. Ibrahem[1,2,3], Mohsin Waris[1], Farzan Shabani[1], Md Rumon Miah[1], Emre Unal [1], Betul Canimkurbey[1,4], Savas Delikanli[1,5,6], and Hilmi Volkan Demir[1,5]*

[1] Department of Electrical and Electronics Engineering, Department of Physics, UNAM − Institute of Materials Science and Nanotechnology, and the National Nanotechnology Research Center, Bilkent University, Ankara 06800, Türkiye.

[2]Laser Science and Technology Department, College of Applied Sciences, University of Technology, Baghdad 10066, Iraq.

[3]Applied Sciences Research Unit, College of Applied Sciences, University of Technology, Baghdad 10066, Iraq.

[4] Department of Physics, Faculty of Polatlı Science and Art, Ankara Hacı Bayram Veli University, Ankara 06900, Türkiye.

[5] Luminous! Center of Excellence for Semiconductor Lighting and Displays, School of Electrical and Electronic Engineering, Division of Physics and Applied Physics, School of Physical and Mathematical Sciences, School of Materials Science and Engineering, Nanyang Technological University, Singapore 639798, Singapore.

[6] Yildiz Technical University, Department of Metallurgical and Materials Engineering, Istanbul 34220, Türkiye.

Corresponding Author

Hilmi Volkan Demir*

Email: volkan@bilkent.edu.tr; hvdemir@ntu.edu.sg

Materials

Selenium (99.99%), 1-octadecene (ODE, 90%), cadmium acetate dihydrate (98%), n-hexane (≥97.0%), toluene (≥99.5%), absolute ethanol, oleic acid (OA, 90%), 2-ethylhexanethiol (EHT, 97%), sodium myristate (≥99.0%), and cadmium nitrate tetrahydrate (≥99.0%) were purchased from Sigma Aldrich.

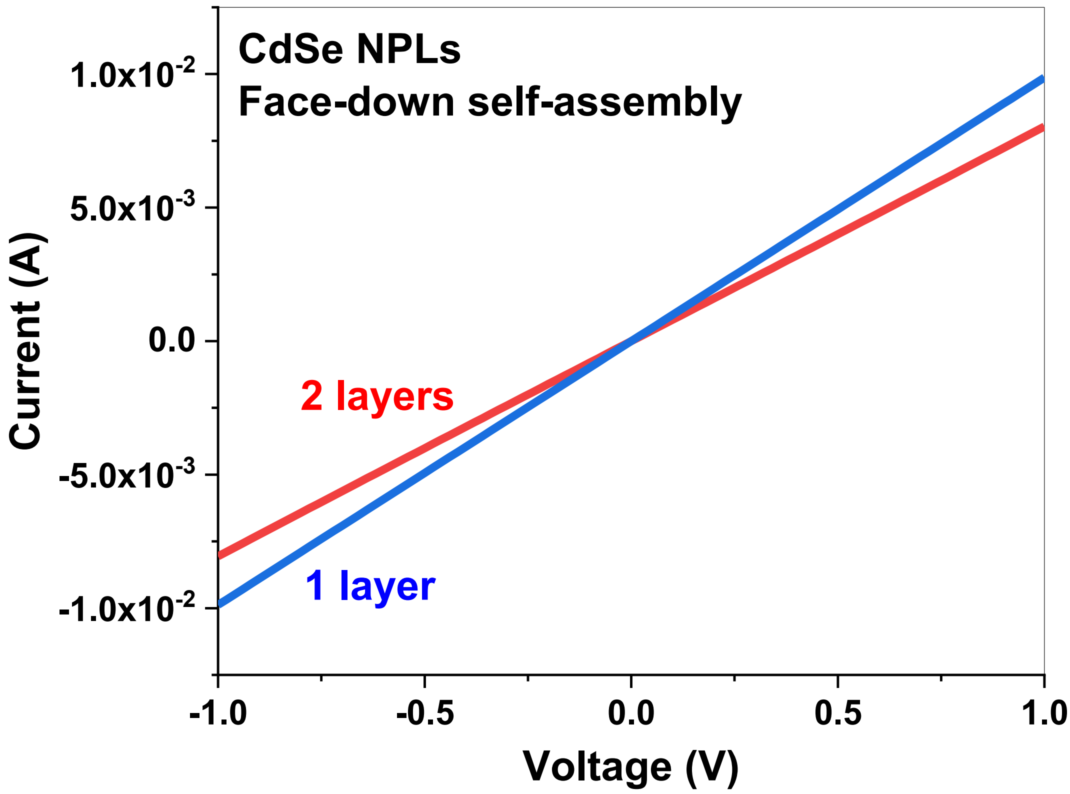

Figure S1. I-V plots of the photodetector device made with 1 and 2 monolayers of face-down assembly of CdSe NPLs in vertical configuration. With one and two FO monolayer assemblies, the device shows short-circuiting between the back Al electrode and the ITO.

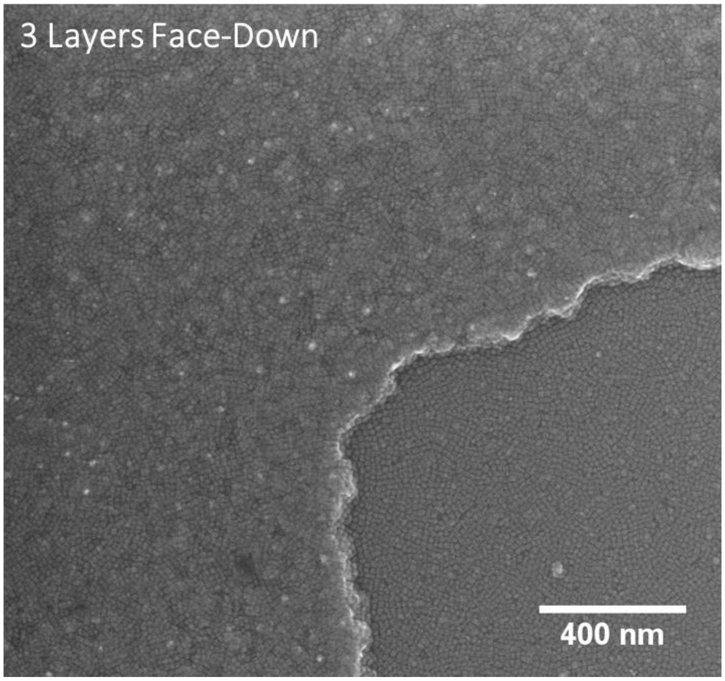

Figure S2. SEM image of the three monolayers of face-down oriented NPLs.

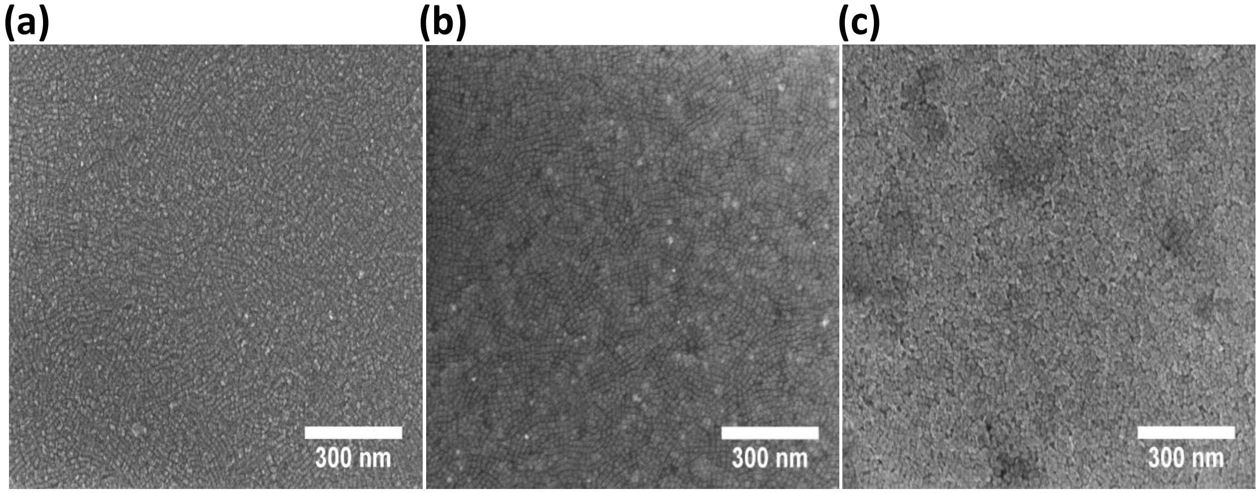

Figure S3. SEM images of the self-assembled NPL layers with (a) EO and (b) 3FO, compared to RO NPL film shown in (c).

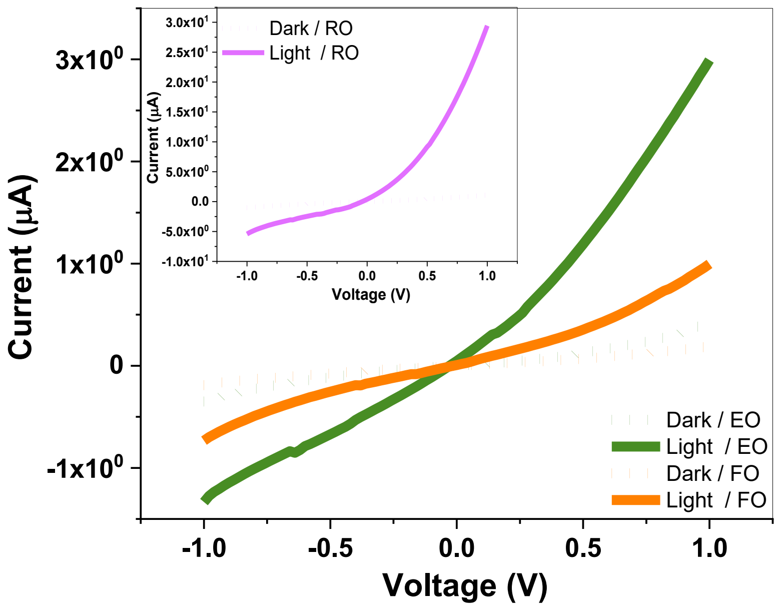

Figure S4: Linear scale I-V plot of the 1EO, 3FO, and RO NPL photodetector devices in dark mode and under LED illumination at 455 nm and 63.42 mW/cm^2 power density.

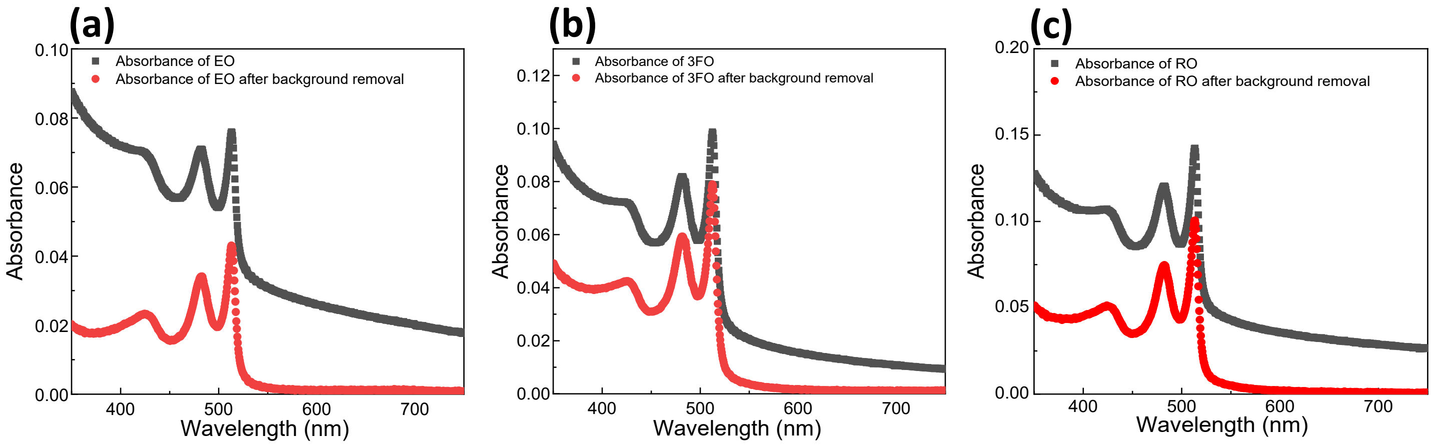

Figure S5: The as-measured and the background-subtracted absorbance spectra comparison of NPL films of (a) edge-up orientation (1EO), (b) 3 layers of face-down orientation (3FO), and (c) random orientation (RO), deposited on a quartz substrate.

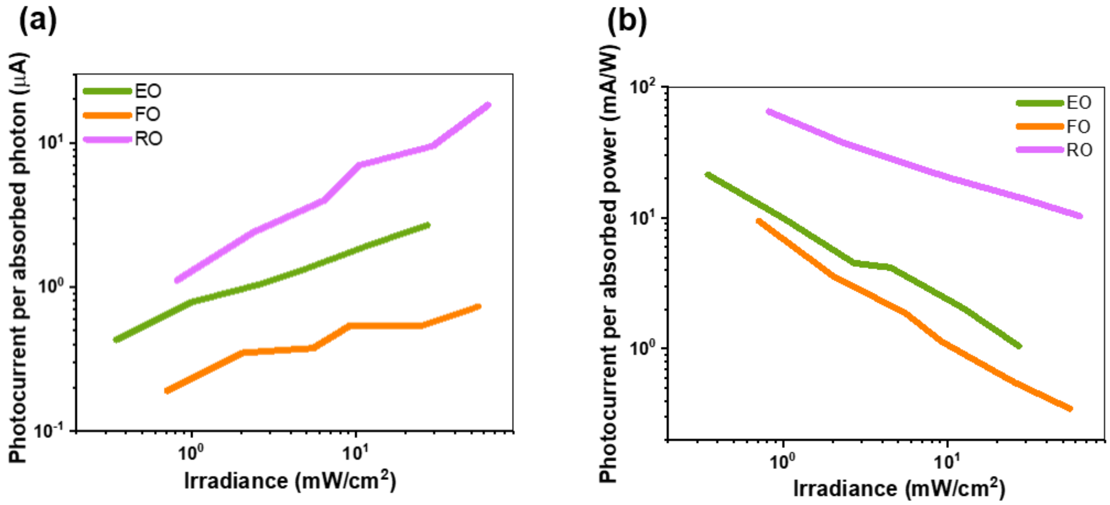

Figure S6: (a) Photocurrent per absorbed photon vs irradiance of the devices illuminated at 455 nm at a bias voltage of 1 V. (b) Photocurrent per absorbed power at a bias voltage of 1 V.

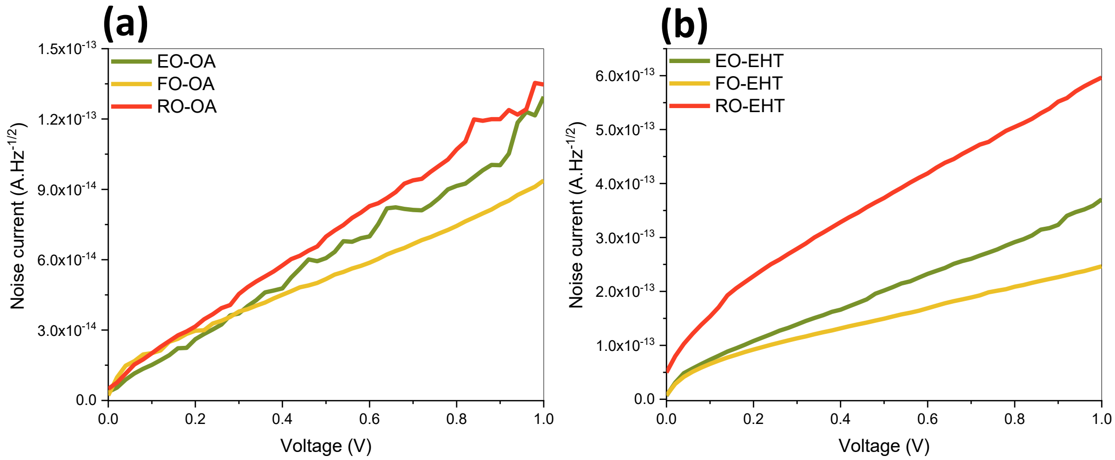

Figure S7: The noise current of CdSe NPLs photodetector devices at different nanocrystal orientations (a) devices with native OA ligands, (b) devices after ligand exchange with EHT.

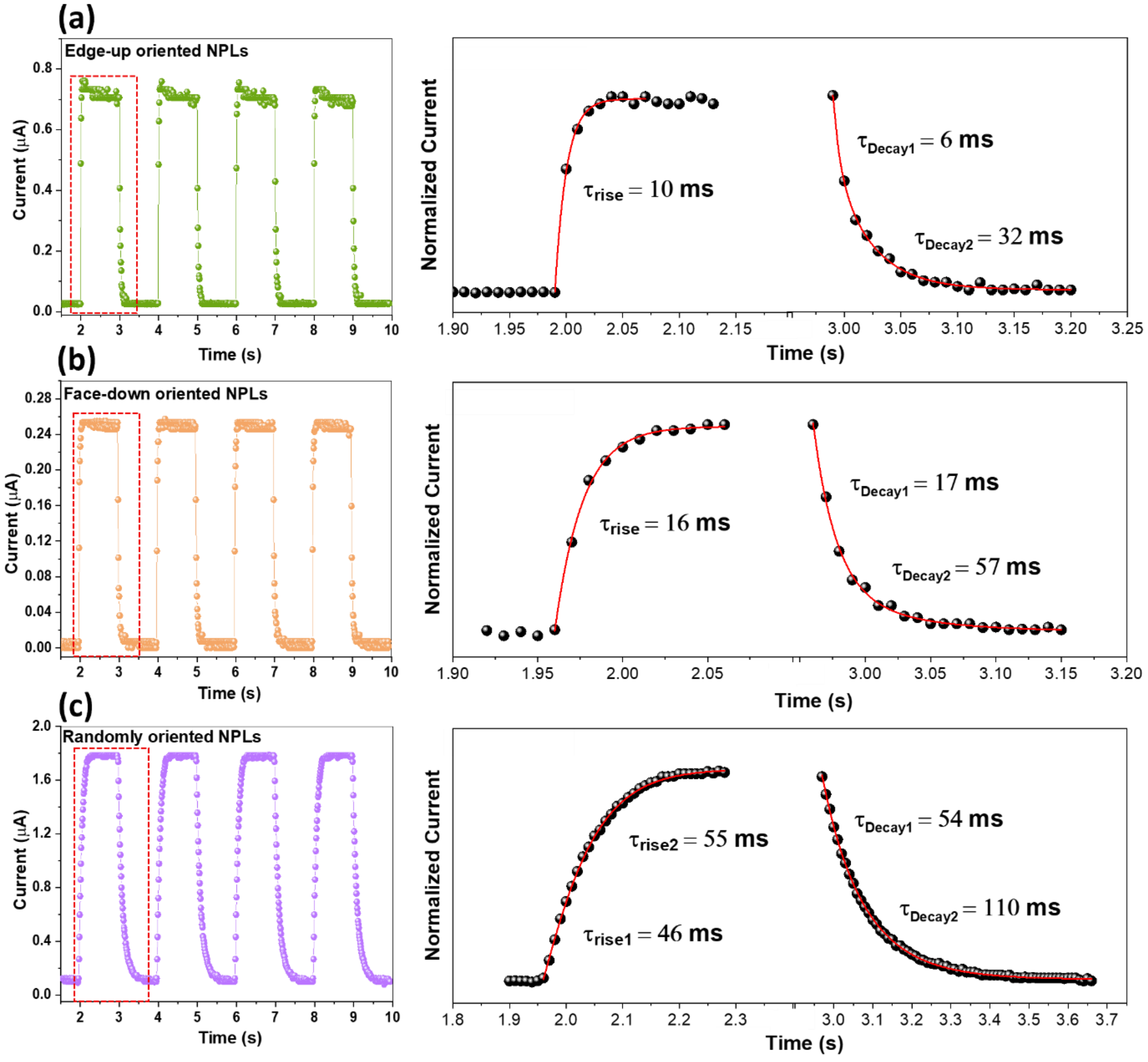

Figure S8: Temporal photoresponse of vertical configuration of the photodetector devices fabricated using various CdSe NPLs with OA ligands in orientations excited with an LED of 455 nm at 0.5 Hz optical cycles with bias voltage of 1 V: (a) EO, (b) FO, and (c) RO.
